# School readiness as a signal of attention-deficit hyperactivity disorder: a population-based cohort study highlighting structural inequalities

**DOI:** 10.1136/archdischild-2025-329285

**Published:** 2025-10-08

**Authors:** Matthew Warburton, Blandine French, Evie Shore, Megan Louise Wood, Mark Mon-Williams

**Affiliations:** 1 School of Psychology, University of Leeds, Leeds, UK; 2 School of Psychology, University of Nottingham, Nottingham, UK; 3 Bradford Institute for Health Research, Bradford, UK

## Abstract

**Objective:**

Assess sociodemographic disparities in attention-deficit hyperactivity disorder (ADHD) diagnoses and determine whether England’s universal assessment of ‘school readiness’ could provide a signal for ADHD-related educational needs.

**Design:**

Population-based cohort study.

**Setting:**

Bradford, UK.

**Method:**

Education and health records were linked using the Connected Bradford database for individuals who completed their first year of compulsory schooling (age 4–5 years) between the 2006/2007 and 2018/2019 academic years (n=1 25 330). School readiness was indexed by the ‘good level of development’ threshold within the Early Years Foundation Stage Profile. Primary healthcare records were used to identify individuals with clinical codes recorded that indicate ADHD diagnosis. Regression analyses allowed controlling for covariates, including sex, ethnicity, free school meals receipt and local area deprivation.

**Results:**

Male and White British heritage individuals were more likely to have received a diagnosis for ADHD than females and those of South Asian heritage or other ethnicities, with the lowest diagnosis rates among South Asian girls. Those who attained a ‘good level of development’ were also less likely to have received a diagnosis for ADHD (attained: 1.0%; not attained: 2.6%, OR=0.40, 95% CI (0.35, 0.45)), even after controlling for covariates (OR=0.42, 95% CI (0.37, 0.48)).

**Conclusions:**

There are structural inequalities in ADHD diagnosis, particularly across sex and ethnicity. Universal school readiness assessments, such as the Early Years Foundation Stage Profile used in England, could act as a signal of ADHD-related educational needs and aid more equitable pathways for ADHD identification and support.

## Introduction

Attention-deficit hyperactivity disorder (ADHD) is a common neurodevelopmental condition characterised by inattention, hyperactivity and impulsivity with a genetic and environmental aetiology.^1 2^ ADHD is commonly diagnosed in childhood but often persists into adulthood^3^ and is associated with a range of negative long-term outcomes including poorer academic attainment, mental and physical health difficulties and reduced quality of life.^4^ These adverse outcomes are seen especially within those who have symptomatic but undiagnosed ADHD,^5 6^ suggesting that early identification and timely support are key to improving a child’s long-term prospects.

However, there are large disparities in the identification, diagnosis and treatment of ADHD. It has been consistently observed that males are more likely to receive a diagnosis for ADHD than females.^7^ This may partly reflect true sex differences, given that diagnoses are typically elevated for males even in community and population samples.^8 9^ However, the greater male-to-female ratio in clinical samples would suggest that there are greater inequalities in receiving a clinical diagnosis for females,^9 10^ and females tend to be diagnosed later.^11 12^ This sex disparity is likely to have several causes, including a lack of understanding of ADHD presentation in females and the attribution of ADHD-related behaviours to other conditions.^13^ There are also notable ethnic disparities in diagnosis, with white individuals more likely to receive a diagnosis than those from minority ethnic backgrounds.^14–16^ Furthermore, while those from low socioeconomic status families are more likely to receive a diagnosis for ADHD, they are less likely to receive treatment and/or support.^17^ Importantly, these disparities have remained despite diagnostic rates for ADHD rising in recent years.^18^

There is a need to identify and support children with ADHD in ways that reduce structural inequalities and allow every child to achieve and thrive within the educational system. One possibility is to make greater use of data generated through education settings, an environment where those with ADHD often struggle. In particular, several countries use ‘school readiness’ measures. For example, state-educated children in England are assessed through the Early Years Foundation Stage (EYFS) Profile during their first year of compulsory education (school Reception year, age 4–5 years). The EYFS comprises a holistic set of ‘early learning goals’ against which teachers assess children’s abilities.^19^ Several of these early learning goals overlap with typical ADHD symptomology, including ‘listening and attention’ and ‘managing feelings and behaviour’. We therefore hypothesised that performance on the EYFS Profile would be related to ADHD diagnoses.

Previous research has demonstrated the utility of the EYFS Profile in identifying children who would benefit from tailored support. For example, research has shown an association between the EYFS Profile and autism diagnoses^20^ and between the EYFS Profile and downstream special educational needs (SEN) support in school.^21 22^ The Profile may offer a less biased way of identifying those with neurodevelopmental conditions, given that teachers are well positioned to assess a child’s needs accurately and holistically. In addition, targeted screening for autism based on low Profile scores appears to offer an effective means of increasing equity in the identification of autism.^23^

In the present work, we sought to assess whether the predictive utility of the EYFS Profile extends to clinical ADHD diagnoses, as identified through primary healthcare records. We also sought to characterise the disparities in clinical diagnosis in a population sample. We used Connected Bradford, a population-scale database collating routinely collected data from individuals across the Bradford district of the UK, to address these topics.^24^

## Methods

### Data

We identified a cohort of individuals (n=1 25 330) who completed the school Reception year (aged 4–5 years) between the 2006/2007 and 2018/2019 academic years (with each year referred to as a ‘school cohort’ hereafter), where the EYFS profile was assessed and where general practice (GP) medical records were available. The data were obtained from Connected Bradford, a database that links administrative records from the citizens of Bradford, UK.^24^ The sample excluded those with multiple EYFS records (n=82); who were not assessed or were exempt from the EYFS assessment^19 25^ (n=266); who did not have complete covariate information including sex, ethnicity, free school meals receipt or a birth year and month available (n=810); or who were diagnosed with ADHD before age 5 years (n=6). We believe these exclusions are unlikely to meaningfully bias the results, given the relatively small proportion of exclusions (~0.9% of the total data) and the similar rates of ADHD among the included (1.4%) and excluded (1.7%) data.

### Measures

#### School readiness

Our binary school readiness measure was whether the individual attained a Good Level of Development (GLD) on the EYFS Profile,^26^ with data provided by the Department for Education. We included a variable for the version of the EYFS Profile completed, as two distinct versions were conducted between 2006/2007–2011/2012 and between 2012/2013–2018/2019.

#### ADHD diagnosis

Individuals diagnosed with ADHD were identified by searching for relevant diagnostic codes across GP records. Specifically, we searched individuals’ records for SNOMED codes^27^ indicative of a diagnosis of ADHD or a synonymous condition (eg, hyperkinetic conduct disorder). The date of extraction for the GP records was 16 May 2025. Prevalence estimates were made by considering the number of individuals with an ADHD diagnosis versus the total number of individuals in a group, with the group either being defined by the school cohort or by levels of demographic factors. Only 0.9% of those with ADHD were diagnosed before 6 years of age, thus a diagnosis of ADHD preceded the EYFS Profile assessment in most cases.

### Covariates

We controlled for a number of covariates on the basis of research showing differential ADHD diagnoses across sociodemographic factors. In particular, we included sex, ethnicity, having ever received free school meals and whether the individual lived in the most deprived quintile of England (ie, lowest 20%) according to the indices of multiple deprivation (IMD) in Reception year. The school cohort was also included to account for transient changes in ADHD diagnoses over time. We coded Ethnicity as ‘White British’, ‘South Asian’ and ‘Other’ ethnicities as a large proportion of Bradford’s population is made up of White British and South Asian heritage individuals. The demographic information for the sample is shown in table 1.

### Statistical analyses

All analyses were performed using R (V.4.4.1). Statistical significance was assessed by comparing p values against a threshold of α=0.05.

ADHD diagnostic rates were first visualised for each school cohort, showing the cumulative percentage of a cohort diagnosed with ADHD across age. We then visualised sex and ethnic disparities in diagnoses for the first and last school cohorts, to track any changes, and also among the whole cohort. We used a likelihood ratio test to check whether an interaction between sex and ethnicity was present by comparing two generalised models with a binomial distribution that either included only the main effects or the main effects and an interaction term.

We then assessed whether school readiness was indicative of those who were diagnosed with ADHD. We found that the EYFS Profile version affected the results, so we present analysis on the newer version of the Profile (2012/2013 onwards). We conducted two logistic regressions with ADHD diagnosis as the outcome. The first regression only used GLD attainment as a predictor. The second regression entered GLD and the covariates. Dummy coding was used for binary and categorical variables so: (1) the intercept reflected the log odds of having an ADHD diagnosis at the reference level of all variables and (2) the coefficients reflected the change associated with moving from the reference level to another level of a given variable (reference levels: sex=female; ethnicity=White British; ever received free-school meals=false, IMD quintile=bottom quintile, school cohort=2012/2013). These results were visualised as ORs by taking the exponent of the regression coefficients. For the full model, around 1% of individuals were missing IMD information, so were deleted case wise. Similar model estimates are obtained when the school during the Reception year is included as a random effect.

## Results

### Trends in ADHD diagnoses

**Figure 1:**
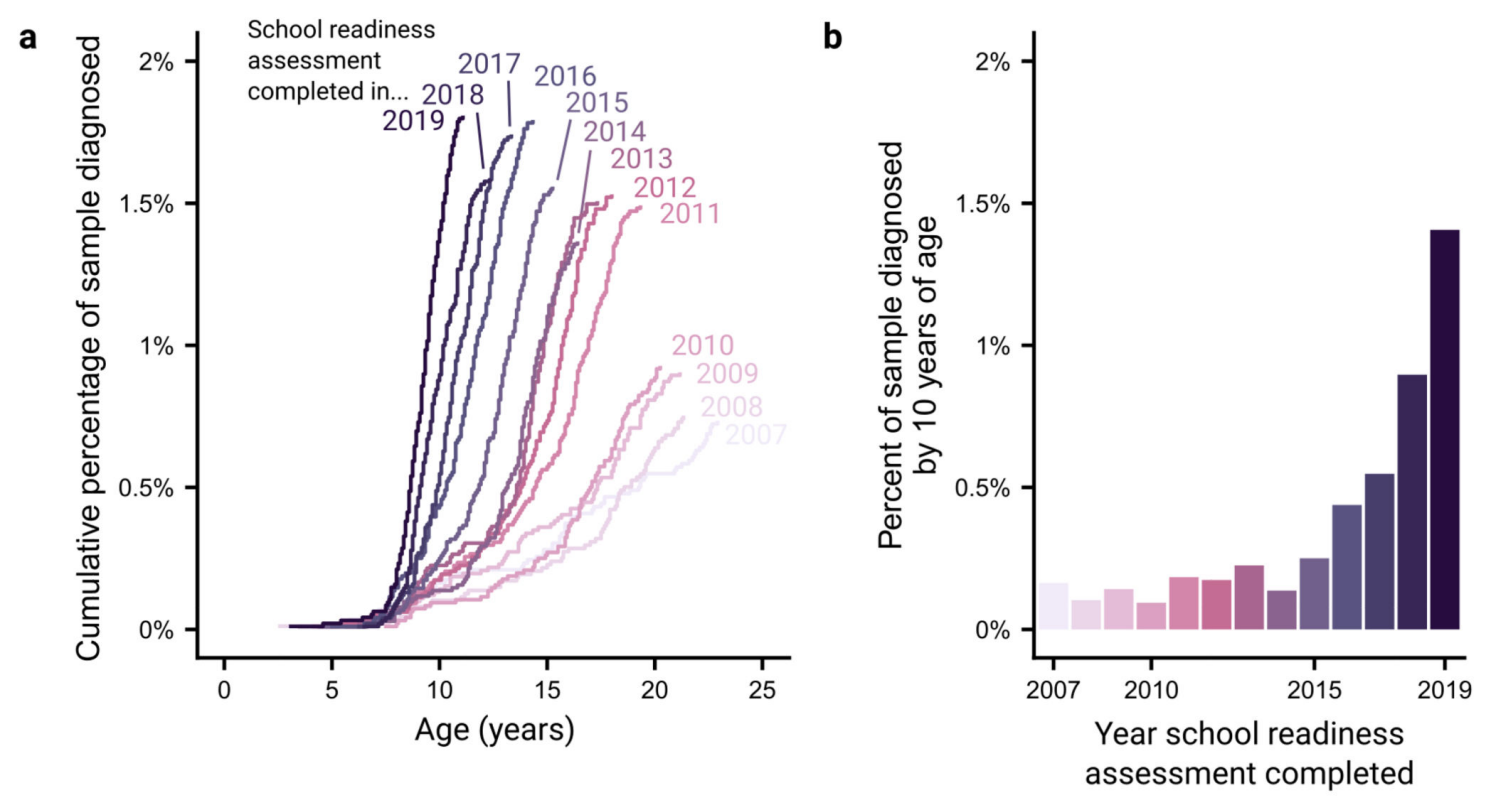
Trends in ADHD diagnoses in recent Bradford school cohorts. (a) Cumulative percent of school cohorts (signified by the year individuals completed Reception year of school) diagnosed with ADHD as their age increases. (b) The percent of each school cohort diagnosed with ADHD by age 10. ADHD, attention-deficit hyperactivity disorder.

We first assessed how the rates of ADHD diagnoses have changed over time. We operationalised this as the cumulative percentage of a given school cohort diagnosed with ADHD by age. We compared these cumulative percentages across school cohorts (figure 1A). We found a stable pattern where ADHD diagnoses generally start being made at around 5–7 years of age. However, it is clear that far more diagnoses were made in more recent school cohorts. To demonstrate this, we extract the cumulative number of individuals diagnosed with ADHD by age 10 years across the different school cohorts (figure 1B). Diagnoses made by age 10 years appear to have risen slowly until 2015, whereafter there has been a rapid rise, with an 8.6 times greater rate for those completing reception year in 2019 compared with those in 2007 (1.41% vs 0.16%, respectively).

### ADHD diagnoses across demographic factors

**Figure 2:**
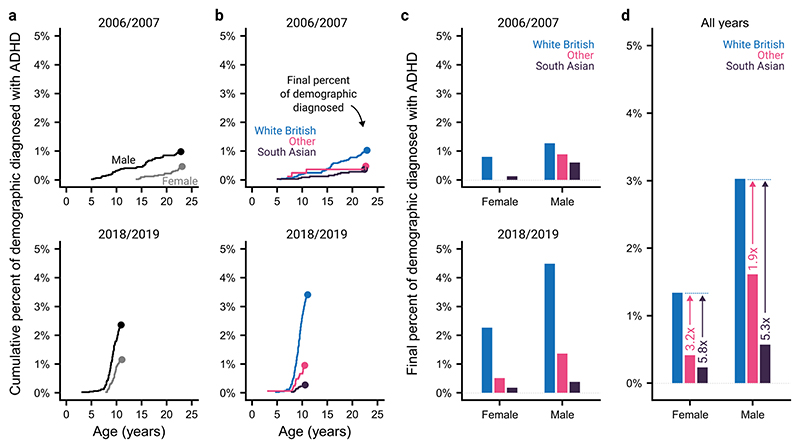
Trends in sex and ethnic differences in ADHD diagnoses. (a) Cumulative percent of male and female individuals in two school cohorts (2006/2007 and 2018/2019) diagnosed with ADHD by age. The dots show the final percent of the demographic diagnosed with ADHD, at the time of the data extract. (b) As (A) but showing ethnic differences. (c) The final percent of individuals diagnosed with ADHD, split by sex and ethnicity for the two school cohorts. Note that there were no ADHD diagnoses for females from ‘other’ ethnicities in the 2007 cohort. (d) As (c) but pooled across all available school cohorts. ADHD, attention-deficit hyperactivity disorder

We next assessed how these trends differ across sex and ethnicity for the first and last school cohorts available. Figure 2A demonstrates that diagnoses are made more frequently for males when compared with females. Inequalities are also evident across ethnicities, with the White British demographic having a higher rate of diagnosis than either South Asians or Other ethnicities. This was true both when the trends in diagnoses with age (figure 2B) or the final percentage of the sample diagnosed at the time of the extract (figure 2C) were considered. These data make it clear that sex and ethnic inequalities do not appear to have appreciably narrowed despite an increase in the overall numbers of diagnoses. However, the low rate of ADHD diagnoses in certain demographics makes their progression over time appear somewhat noisy.

To gain a more precise understanding of these inequalities, we pooled data over all the available years (figure 2D). These pooled data show that, across all ethnicities, there is a higher incidence of ADHD diagnoses for males compared with females, and that within both sexes, South Asian and individuals of other ethnicities are diagnosed less frequently than White British individuals. Further, this ethnic inequality is more pronounced for females, demonstrating a double disadvantage (ratio between incidence in White British and South Asian individuals: 5.3× for males, 5.8× for females; ratio between incidence in White British and other ethnicities: 1.9× for males, 3.2× for females). This was supported by a likelihood ratio test that indicated the presence of an interaction between sex and ethnicity (χ^2^=8.32, p=0.016).

### Does school readiness signal ADHD diagnoses?

We finally assessed whether reaching a GLD on the EYFS Profile, conducted on all state-educated pupils in England, was associated with receiving an ADHD diagnosis. Across the whole sample, those who attained a GLD were less likely to have received an ADHD diagnosis (1.9% vs 0.9%; OR=0.47, 95% CI (0.43, 0.52), p<0.001). However, there was a significant interaction between GLD attainment and the version of the Early Years Foundation Stage profile conducted (OR=0.70, 95% CI (0.57, 0.86), p<0.001), such that achieving a GLD on the pre-2013 version was more weakly associated with an ADHD diagnosis (before 2012/2013: 1.4% vs 0.8%; 2012/2013 onwards: 2.6% vs 1.0%). We therefore present analyses for only those who completed reception year in or after the 2012/2013 academic year (but note that all inferences were consistent for the other version unless highlighted otherwise).

**Figure 3:**
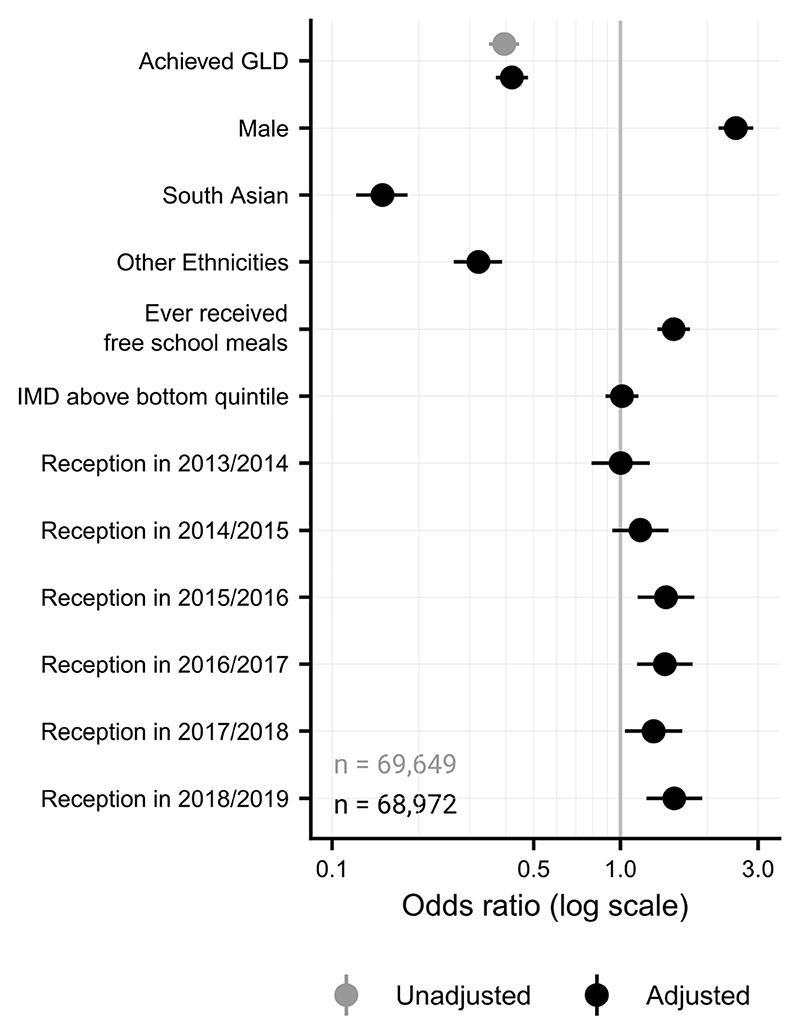
ORs for logistic regressions assessing ADHD diagnoses. The unadjusted model, where only GLD achievement was entered, is shown in grey, whereas the full adjusted model, which included all covariates, is shown in black. The X-axis is displayed on the log scale, with a value of 1 reflecting no effect on ADHD diagnosis. Restricted to those who completed Reception year during or after 2012/2013, where the Early Years Foundation Stage profile changed. OR, odds ratio; ADHD, attention-deficit hyperactivity disorder; GLD, Good Level of Development; IMD, indices of multiple deprivation

For the unadjusted regression, where only GLD attainment was entered, achieving a GLD was associated with significantly lower chances of receiving an ADHD diagnosis (OR=0.40, 95% CI (0.35, 0.45), p<0.001; figure 3). This held even after adjusting for covariates (OR=0.42, 95% CI (0.37, 0.48), p<0.001). Males were more likely to receive a diagnosis, and those from South Asian or other ethnic backgrounds were less likely to receive an ADHD diagnosis. Further, those having ever received free school meals were more likely to receive an ADHD diagnosis (though this was not the case for the pre-2012/2013 sample), as were those completing reception in all years from 2014/2015 to 2018/2019, but living in an area associated with an index of multiple deprivation above the bottom quintile was not significantly associated with ADHD diagnoses. These results were consistent when the total score on the EYFS Profile was used instead.

## Discussion

We have demonstrated—at a population level—that there are large disparities in clinical ADHD diagnoses as a function of sociodemographic factors. These disparities do not appear to have decreased appreciably with time despite an overall increase in diagnostic rates. Further, we have shown that England’s universal assessment of ‘school readiness’ can identify children who are likely to have educational needs related to ADHD.

Our use of the Connected Bradford database^24^ allowed us to investigate the disparities in ADHD diagnoses in a highly diverse population. Our results demonstrated that White British individuals were more likely to have an ADHD diagnosis than either South Asian individuals or individuals from other ethnic backgrounds. This is consistent with a range of prior research showing lower diagnosis rates among ethnic minorities,^14–16^ though often in the context of the USA. Several reasons have been offered for these ethnic disparities, including cultural differences in the recognition of problem behaviours, fear of stigmatisation, limited knowledge of ADHD and barriers to navigating the referral pathways.^28–30^ We also found that females were less likely to be diagnosed than males, consistent with prior clinical studies.^11 12^ Further, the intersection of these two factors meant that female ethnic minorities received the lowest diagnosis rates, aligning with previous work showing intersectionality in ADHD diagnosis.^31^ Future work could take a qualitative approach to understand the barriers to ADHD diagnoses along different social dimensions, especially in the unique context of Bradford where a traditional minority ethnicity (South Asian) makes up a large proportion of the population.

We also demonstrated that attainment of a ‘good level of development’ on England’s universal assessment of school readiness was related to a lower probability of a clinical diagnosis of ADHD. Performance on the EYFS Profile is associated with downstream autism diagnosis and support for SEN,^20–22^ suggesting school readiness measures may offer a means to aid identification of those who would benefit from support more broadly. Further, the Profile’s association with negative downstream consequences such as poor academic attainment and a ‘Not in Education, Employment or Training’ outcome^32^ would suggest it captures a broad range of factors important for successful school trajectories.

Previous work has shown that targeted autism screening based on EYFS Profile scores can aid the identification of those with autism.^23^ The current findings suggest the EYFS Profile may also be useful for aiding early and equitable identification and support of ADHD learning needs. It is of note that only a modest percentage of those who failed to attain a GLD went on to receive an ADHD diagnosis (2.6% on the new version). This percentage is likely to rise with systematic screening, but the sensitivity of the measure would likely remain low and would not reflect a cost-effective screening criterion alone. It is possible that a subset of the EYFS assessment might increase sensitivity, given that some of the domains align more strongly with ADHD symptomology than others. However, the EYFS Profile would likely be most effective as a clinical screening tool when combined with other public service measures. These measures could potentially trigger a multi-service assessment of whether the child would benefit from a specialist evaluation. We note that a better approach might be to use the EYFS Profile to identify need and, in the first instance, explore whether that need can be addressed within the educational setting. If the child’s needs cannot be met, then a referral with this information would provide an efficient clinical pathway.

There are several limitations to this study. One issue is that the reported data are from the Bradford district, an area with relatively higher diversity than other areas of England which may influence the barriers and pathways to assessment. Further, the current work used clinical diagnoses as the outcome measure, but the study itself shows this outcome is subject to demographic disparities. Thus, the study does not provide any direct evidence as to whether the EYFS can increase equity in diagnoses. Future work could address this issue by taking a similar approach as used previously regarding autism assessments.^23^ Relatedly, there is evidence that teacher-based assessments are subject to similar disparities across sex and ethnicity,^33–35^ and factors beyond the individual’s abilities may be used to moderate scores on the assessment.^36 37^ The extent to which using EYFS Profile scores would introduce different biases to the assessment pathways is therefore unclear. Finally, we were only able to control for a limited set of covariates. Among these, the indicator for having ever received free school meals is likely to underestimate the number of disadvantaged pupils, as more individuals are eligible for free school meals than those who actually apply.^38^

In conclusion, we have confirmed that large disparities exist in ADHD diagnoses across sex and ethnicity in a highly diverse population sample. We have demonstrated that England’s universal assessment of school readiness is associated with downstream clinical ADHD diagnoses. The EYFS measure is already available for all state-educated children in England, so that it could offer a cost-effective means to aid early identification of ADHD when combined with other measures. This approach would allow appropriate support to be provided to children and young people and help them achieve and thrive in educational settings.

## Figures and Tables

**Table 1:** Demographic information for the sample

	Full sample	No ADHD diagnosis	ADHD diagnosis
n	125 330	123 603	1727
ADHD			
No ADHD diagnosis	123 603 (98.6%)	123 603 (100%)	0 (0%)
ADHD diagnosis	1727 (1.4%)	0 (0%)	1727 (100%)
GLD			
Not attained	55 097 (44.0%)	54 024 (43.7%)	1073 (62.1%)
Attained	70 233 (56.0%)	69 579 (56.3%)	654 (37.9%)
Gender			
Female	60 735 (48.5%)	60 249 (48.7%)	486 (28.1%)
Male	64 595 (51.5%)	63 354 (51.3%)	1241 (71.9%)
Ethnicity			
White British	61 135 (48.8%)	59 784 (48.4%)	1351 (78.2%)
South Asian	46 009 (36.7%)	45 822 (47.1%)	187 (10.8%)
Other	18 186 (14.5%)	17 997 (14.6%)	189 (10.9%)
Ever received FSM			
False	76 997 (61.4%)	76118 (61.6%)	879 (50.9%)
True	48 333 (38.6%)	47 485 (48.4%)	848 (49.1%)
IMD above bottom quintile			
False	57 391 (45.8%)	56 636 (45.8%)	755 (43.7%)
True	48 819 (39.0%)	47 996 (38.8%)	823 (47.7%)
(Missing)*	19 120 (15.3%)	18 971 (15.3%)	149 (8.6%)
Academic year			
2006/2007	8572 (6.8%)	8509 (6.9%)	63 (4.6%)
2007/2008	8769 (7.0%)	8703 (7.0%)	66 (3.8%)
2008/2009	9168 (7.3%)	9085 (7.4%)	83 (4.8%)
2009/2010	9592 (7.7%)	9504 (7.7%)	89 (5.2%)
2010/2011	9780 (7.8%)	9634 (7.8%)	146 (8.5%)
2011/2012	9796 (7.8%)	9646 (7.8%)	150 (8.7%)
2012/2013	10 216 (8.2%)	10 061 (8.1%)	155 (9.0%)
2013/2014	10 250 (8.2%)	10109 (8.2%)	141 (8.2%)
2014/2015	10 395 (8.3%)	10 233 (8.3%)	162 (9.4%)
2015/2016	9591 (7.7%)	9419 (7.6%)	172 (10.0%)
2016/2017	10 043 (8.0%)	9868 (8.0%)	175 (10.1%)
2017/2018	9702 (7.7%)	9548 (7.7%)	154 (8.9%)
2018/2019	9455 (7.5%)	9284 (7.5%)	171 (9.9%)

Raw counts and percentage of the relevant sample (column) are given per level of each variable.

* The vast majority of the missingness occurred because no IMD information was available for those in academic years 2006/2007 and 2007/2008. FSM, free school meal; GLD, Good Level of Development; IMD, indices of multiple deprivation.

## Data Availability

Data may be obtained from a third party and are not publicly available. The study uses data from the Connected Bradford database, a set of linked administrative datasets, which can be accessed by bona fide researchers through an application to use the data. The scripts used to analyse the data are available at https://osf.io/rcx83.

## What is Already Known on this Topic

⇒ Clinical diagnoses for attention-deficit hyperactivity disorder (ADHD) are made less often for females and those from ethnic minorities.
⇒ England’s school readiness measures are associated with downstream special educational needs and have the potential to increase the equity of autism identification and support.

## What This Study Adds

⇒ The ethnic, sex and socioeconomic disparities observed in clinical ADHD diagnoses reflect patterns found in a highly diverse population sample.
⇒ School entry assessments can signal ADHD-related educational needs.

## How This Study Might Affect Research, Practice Or Policy

⇒ School entry assessments could be used to facilitate earlier identification of ADHD need and reduce inequalities in the identification and support of neurodevelopmental conditions.
